# Evaluation of Demarcation Line after Epithelium-Off Iontophoresis Corneal Collagen Cross-Linking for Progressive Keratoconus

**DOI:** 10.3390/jcm10153295

**Published:** 2021-07-26

**Authors:** Francesco D’Oria, Pasquale Puzo, Cosimo Incandela, Alessandra Sborgia, Samuele Gigliola, Francesco Boscia, Giovanni Alessio

**Affiliations:** Section of Ophthalmology, Department of Basic Medical Sciences, Neurosciences and Sense Organs, University of Bari, 70124 Bari, Italy; puzopasquale@gmail.com (P.P.); cosimo.incandela1993@gmail.com (C.I.); alessandrasborgia@yahoo.it (A.S.); samuelegigliola@yahoo.it (S.G.); francescoboscia@hotmail.com (F.B.); giovanni.alessio@uniba.it (G.A.)

**Keywords:** keratoconus, corneal collagen cross-linking, iontophoresis, anterior segment, optical coherence tomography

## Abstract

The aim of the study was to visualize and evaluate the characteristics and depth of the demarcation line with anterior segment optical coherence tomography (AS-OCT) after epithelium-off iontophoresis corneal collagen cross-linking (epi-off I-CXL). In this prospective, consecutive, single center study 18 eyes of 18 patients with keratoconus were involved. One month after epi-off I-CXL, all the patients underwent an AS-OCT scan to search for a demarcation line and its characteristics. The corneal stromal demarcation line was identified in all the eyes. Mean depth of the corneal stromal demarcation line was 261.8 ± 46.7 μm (range: 184 to 362 μm), at 56.7 ± 12% corneal depth. In conclusion, epi-off I-CXL determines a demarcation line that can be visualized with AS-OCT, which seems clearly distinguishable and similar to that created in standard CXL.

## 1. Introduction

Keratoconus is a chronic and progressive disease involving the cornea: the biomechanical instability of the stroma causes its thinning and deformation, so the cornea assumes the shape of a cone, losing its characteristic rigidity and resistance. Corneal involvement is usually bilateral and asymmetrical, affecting up to 1:375 people in some populations [1]. Disease detection is essential for improving the management of keratoconus patients as it can advance from mild changes to a severe loss of visual acuity [2], which might require corneal transplantation because advanced disease with severe thinning and ectasia and eventually corneal scarring would not be further managed by other optical corrections [3]. In the last decades, the corneal collagen cross-linking (CXL) procedure has made it possible to stabilize or at least slow down the progress of those forms of progressive keratoconus, reducing the number of corneal transplants [4]. The procedure allows, through the impregnation of the cornea with vitamin B12 and the subsequent irradiation with ultraviolet UVA rays, to increase the formation of covalent bonds between the fibers of the innermost layers of the corneal stroma, making it more resistant and limiting corneal thinning and subsequent progression of keratoconus [5]. The standard method of epithelium off CXL, referred to as “Dresden protocol”, implies the preventive removal of the corneal epithelium to allow the penetration of riboflavin inside the corneal stroma [4]. Iontophoresis allows the penetration of riboflavin into the corneal stroma using electric current [6]. This method was initially used to allow the penetration of an adequate amount of riboflavin inside the corneal stroma but maintaining the corneal epithelium [7,8]: a 5-min iontophoresis protocol achieves a riboflavin concentration in the corneal stroma sufficient for CXL treatment with the advantage of shortening the imbibition time [8]. Since 2 weeks after the treatment, it is possible to identify a demarcation line on slit-lamp [9], confocal microscopy [10] and anterior segment optical coherence tomography (AS-OCT). This demarcation line appears on the AS-OCT imaging as a hyper-reflective line in the corneal stroma, at a variable depth [11]. Its presence, and the depth that the line reaches at the stromal level, might be considered as an indirect predictor of the effectiveness of the treatment as it represents the transition between the anterior treated stroma and the posterior untreated stroma; various studies are comparing the effectiveness of different CXL protocols based on this indirect parameter [11,12,13]; nevertheless, Yam et al. failed to find a correlation between demarcation line depth and change in visual acuity and in steepest K at 6 months [14]. The aim of the study is to visualize and evaluate the characteristics of the demarcation line one month after a novel CXL protocol named epithelium-off iontophoresis CXL (epi-off I-CXL) as assessed by AS-OCT.

## 2. Materials and Methods

### 2.1. Patients

Prospective, single center study that included 18 consecutive patients (regardless the sex) with progressive keratoconus, classified according to the Amsler–Krumeich grading system. The diagnosis was based on corneal topography data obtained by Sirius (Costruzione Strumenti Oftalmici, Firenze, Italy) and slit-lamp examination. Inclusion criteria were progressive keratoconus (defined as progressive when there was an increase in Kmax of at least 1.o Diopter in 1 year) and corneal thickness greater than 400 μm. Exclusion criteria were any history of intraocular or corneal surgery, central corneal opacities, pregnancy, lactation, and any other corneal pathology; patients with a maximum K steeper than 61 D were also excluded. AS-OCT scan measurements were performed by two independent examiners (FD and PP) at 1-month post-operative with Huvitz OCT to visualize the demarcation line and define its depth (in μm and in percentage of total corneal thickness). All procedures were conducted in accordance with the ethical standards established by the Declaration of Helsinki.

### 2.2. Surgical Technique

All the surgical procedures were performed by two surgeons experienced in the field (FD and PP) under sterile conditions. Before surgery, topical anesthesia (oxybruprocaine hydrochloride 0.4% eye drops) was instilled, and the corneal epithelium was mechanically debrided over a 9 mm area from the center of the cornea. The iontophoresis device (Iontofor CXL; SOOFT Italia S.p.A., Montegiorgio, Italy) was filled with 0.1% riboflavin (Ricrolin+; SOOFT Italia S.P.A.); the iontophoresis system was set at 1.0 mA and applied for 5 min. After the iontophoretic procedure, the corneal applicator was removed from the cornea. Ultraviolet-A irradiation was then performed using a commercially available ultraviolet-A optical system (C.B.M. X-Linker Vega 10 mW; CSO, Florence, Italy). Irradiance was performed for 9 min, corresponding to a total surface dose of 5.4 J/cm^2^.

Postoperative medication included topical netilmicin and dexamethasone drops three times per day for 1 weeks followed by topical dexamethasone drops for 3 weeks more, cycloplegic eye drops two times per day for 2 days, and topical lubricants for 1 month.

### 2.3. Statistical Analysis

Patient’s data and clinical data were gathered and organized in a Microsoft Office Excel file. The data were analyzed using SPSS for Windows software (version 22.0, SPSS, Inc., IBM, Armonk, NY, USA). Quantitative data were described using mean and standard deviation, as well as minimum and maximum.

## 3. Results

A total of 18 eyes of 18 patients with a mean age of 21 ± 4 years (range 13 to 29) were included. Pre-operative corneal thickness was 479 ± 24.7 μm (range: 402 to 570 μm). The mean central corneal thickness measured with AS-OCT at 1-month post-operative was 466 ± 41.9 μm (range: 400 to 559 μm). The visualization, if yes or no, and depth of the demarcation line in µm and percentage with AS-OCT are presented in Table 1. Mean depth of the corneal stromal demarcation line was 261.8 ± 46.7 μm (range: 184 to 362 μm), at 56.7 ± 12% corneal depth.

**Figure 1:**
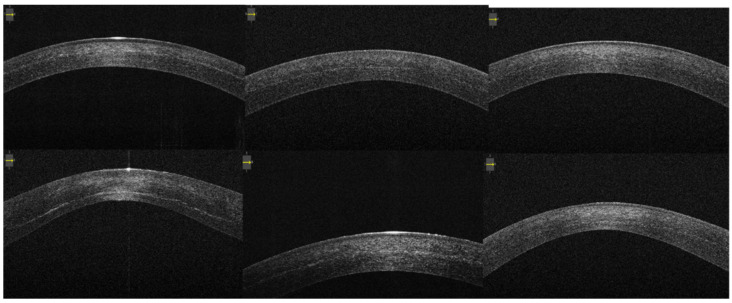
Anterior segment optical coherence tomography visualizing demarcation lines in a sample of patients 1 month after iontophoresis-assisted epithelium-off corneal collagen cross-linking.

The corneal stromal demarcation line was identified in all the patients by both examiners. It appears as a well-defined hyper-reflective line in the posterior corneal stroma at a variable depth between 50–70% (Figure 1).

We have not observed any intraoperative or postoperative complications such as infectious keratitis, persistent stromal haze or delayed epithelial defect in any of the patients.

## 4. Discussion

The goal of this study has been to evaluate the demarcation line of a novel protocol of CXL one month after treatment using AS-OCT to visualize and study the demarcation line and its depth. In our study, we are using an epithelium-off iontophoresis-assisted CXL protocol: mediated iontophoresis imbibition for 5 min and an irradiation of 10 mW/cm^2^ for a duration of 9 min were performed. This protocol led to a good, clearly distinguishable demarcation line in all patients, at an average depth of 50–70% of the corneal thickness.

Numerous studies have demonstrated the effectiveness of the epi-off standard CXL, also called ‘Dresden protocol’, in progressive forms of keratoconus [4,15,16,17]: after removing the corneal epithelium, it requires 30 min of soaking with vitamin B12 and UVA irradiation 3 mW/cm^2^ for 30 min, for a total dose of 5.4 J/cm^2^. Therefore, the standard method takes about 60 min to complete the entire procedure, with related costs for the staff employed and stress for the patient undergoing the procedure. The reduction of the duration of treatment, maintaining the same efficacy [18,19], in recent years, has been the focus of various protocols, that is to say, there are various studies that aim at reducing the penetration time of the right quantities of vitamin B12 to the adequate thickness of the corneal stroma. One of the most recently used is the iontophoresis protocol, which allows faster imbibition of the corneal stroma with riboflavin, thus shortening the entire procedure when compared to the standard Dresden protocol. The possibility of preserving the corneal epithelium while maintaining the same effectiveness of the epi off methods has however been questioned by the results of several studies [11,18].

The need to compare the results obtained with the different protocols has required the setting of parameters apt to predict if the treatment is successful or not, as the changes in best-corrected visual acuity and K readings [20] or the changes in corneal density [21]. A parameter that today seems to satisfy the need of indirect predictor of effectiveness is the demarcation line [9], and various studies compare the effectiveness of different cross-linking protocols based on the demarcation line [11,12,13,22]. Several authors believe that its identification and the depth that the line reaches at the stroma level are a predictor of the effectiveness of the treatment because, indeed, the stroma seems to be more resistant after the treatment [8]. However, not all studies confirm this thesis [23,24], stating that no correlation has been found between the depth of the DL and the change in K values [23] and hypothesizing that this dividing line may be only a sign of non-specific inflammation [24]. It is possible that the deeper the demarcation line, the more stroma is treated, and thus, the treatment can be more effective; however, the correlation of demarcation line depth and treatment effectiveness in terms of arrest of disease progression has not been yet demonstrated.

Vinciguerra et al. using the same protocol of our study identified a non-statistically significant difference between the standard and epi-off I-CXL protocols in most of the parameters, including visual acuity, topographic indexes and maximum keratometry after 2 years [19]. In our study as well a good, well distinguishable and dividing line is visible in all patients, at an average depth of 56.7% of the corneal thickness (range 42 to 84%) with a reduction of the overall time treatment (15 min) compared to the standard CXL protocol (60 min).

Limitations of the study are the small study samples and the absence of a control group. Given the small number of works related to this new protocol, only further studies, with larger samples and longer follow-up times will confirm the long-term effectiveness of this promising and less time-consuming protocol.

## Figures and Tables

**Table 1:** Depth of visualization of demarcation line after iontophoresis-assisted epithelium-off corneal collagen cross-linking by anterior segment optical coherence tomography.

Eye	Sex	Age	Demarcation Line Visualization	Demarcation Line Depth, µm (%)
1	Male	20	Yes	219 (48%)
2	Male	20	Yes	273 (49%)
3	Male	22	Yes	236 (52%)
4	Male	22	Yes	230 (51%)
5	Male	21	Yes	291 (73%)
6	Male	22	Yes	184 (42%)
7	Male	26	Yes	297 (74%)
8	Male	13	Yes	362 (84%)
9	Male	29	Yes	223 (50%)
10	Male	21	Yes	222 (45%)
11	Male	25	Yes	272 (56%)
12	Male	26	Yes	302 (58%)
13	Male	26	Yes	257 (58%)
14	Male	23	Yes	282 (59%)
15	Female	15	Yes	337 (69%)
16	Male	18	Yes	223 (50%)
17	Female	20	Yes	238 (47%)
18	Female	18	Yes	252 (54%)

## Data Availability

The data presented in this study are available on request from the corresponding author. The data are not publicly available due to privacy.
